# Renal mass biopsies: time to tailor the approach

**DOI:** 10.1007/s00330-025-12067-1

**Published:** 2025-10-16

**Authors:** Annemarie Uhlig

**Affiliations:** Department of Urology, University Medical Center Goettingen, Goettingen, Germany

Renal mass biopsy (RMB) has long been recognised as a valuable diagnostic tool for small renal masses (SRMs), enabling histology-guided management and reducing the risk of overtreatment [1]. Although current clinical guidelines advocate for the use of RMB, utilisation rates remain modest [2, 3]. This is partly attributable to persistent concerns in the literature regarding non-diagnostic outcomes and procedural uncertainties [4].

One key procedural consideration is the choice between coaxial and sequential biopsy techniques. This remains a matter of debate, particularly with respect to diagnostic performance, complication rates, and the theoretical risk of tumour seeding. In this context, García et al address an important evidence gap by conducting the largest direct comparative analysis to date between these two techniques [5].

Drawing upon data from 1174 RMB procedures over a 16-year period, the authors provide a detailed evaluation of diagnostic accuracy, procedural safety, and oncological outcomes. Their analysis demonstrated no statistically significant difference in diagnostic yield between the coaxial (87%) and sequential (88%) approaches. While unadjusted complication rates were higher in the coaxial group, this difference lost statistical significance following multivariable adjustment for imaging modality. CT guidance was more frequently used alongside the coaxial technique, likely influencing complication rates. This analytical nuance represents a key strength of the study, underscoring the importance of contextual factors beyond biopsy technique alone.

Tumour seeding, though rare, remains a concern in RMB practice. Despite recommendations from the European Association of Urology (EAU) favouring coaxial biopsy to mitigate this risk, empirical evidence is limited. In the current study, only two cases (< 0.2%) of histologically confirmed tumour seeding were observed, one with each technique. Both involved papillary renal cell carcinoma, and neither case resulted in local recurrence during follow-up. These findings align with previous literature indicating that tumour seeding is exceptionally rare and not demonstrably preventable by using a coaxial sheath.

A further strength of this work is the evaluation of the rate of pathological upstaging from clinical T1a to pathological T3a, which was low (5%) and did not differ significantly between techniques. Similarly, no differences were observed in surgical margin status or recurrence rates. These real-world outcome measures reinforce the authors’ conclusion that both biopsy techniques are diagnostically reliable and oncologically safe.

While recognising the inherent limitations of the retrospective, single-centre design, the study findings should be interpreted as hypothesis-generating rather than guideline-challenging.

Yet, the findings advocate for an individualised approach to biopsy technique selection, taking into account lesion characteristics, operator expertise, imaging modality, and institutional resources. Such flexibility may lower barriers to RMB adoption in centres predominantly using sequential techniques and could facilitate broader implementation of RMB in the management of SRMs.

Nevertheless, unanswered questions remain. While the authors report median follow-up duration, long-term oncological outcomes in non-surgically managed patients (e.g., those undergoing ablation or active surveillance) were not fully addressed, largely due to treatment heterogeneity and the constraints of retrospective design. Future multicentre prospective studies are needed to validate the results across diverse clinical settings and populations.

## Conclusion

This study provides timely and robust evidence that challenges the presumed superiority of the coaxial technique in RMB. While not calling for its discontinuation, the findings support a more nuanced, individualised approach to technique selection. Importantly, the extremely low incidence of tumour seeding—irrespective of technique—should reassure both clinicians and patients regarding the safety of RMB when performed by experienced operators.
